# Drug metabolizing enzyme activities versus genetic variances for drug of clinical pharmacogenomic relevance

**DOI:** 10.1186/1559-0275-8-12

**Published:** 2011-07-28

**Authors:** Alan HB Wu

**Affiliations:** 1 Department of Laboratory Medicine, University of California, San Francisco, San Francisco General Hospital, 1001 Potrero, San Francisco CA 94110, USA

## Abstract

Enzymes are critically important in the transportation, metabolism, and clearance of most therapeutic drugs used in clinical practice today. Many of these enzymes have significant genetic polymorphisms that affect the enzyme's rate kinetics. Regarding drug metabolism, specific polymorphisms to the cytochrome (CYP) P450 enzyme family are linked to phenotypes that describe reaction rates as "ultra", "intermediate", and "poor," as referenced to "extensive" metabolizers that are assigned to wildtype individuals. Activity scores is an alternate designation that provides more genotype-to-phenotype resolution. Understanding the relative change in enzyme activities or rate of clearance of specific drugs relative to an individual's genotypes is an important component in the interpretation of pharmacogenomic data for personalized medicine. Currently, the most relevant drug metabolizing enzymes are CYP 2D6, CYP 2C9, CYP 2C19, thiopurine methyltransferase (TPMT) and UDP-glucuronosyltransferase (UGT). Each of these enzymes is reactive to a host of different drug substrates. Pharmacogenomic tests that are in routine clinical practice include CYP 2C19 for clopidogrel, TPMT for thiopurine drugs, and UDP-1A1 for irinotecan. Other tests where there is considerable data but have not been widely implemented includes CYP 2C9 for warfarin, CYP 2D6 for tamoxifen and codeine, and CYP 2C19 for the proton pump inhibitors.

## 1. Introduction

Pharmacogenomics is an important tool for the personalization of medical therapeutics. The determination of an individual's genotype for key enzymes that participate in the transportation, metabolism, and clearance is a strong determinant of therapeutic efficacy and toxicity avoidance. Regarding drug metabolism, subjects who have no or slower rate of enzyme activity than normal have higher circulation drug concentrations and are vulnerable to toxicity when the standard dosage is used. Individuals who have a faster of enzyme activity have lower circulating drug concentrations and may be sub-therapeutic at standard dosage. The situation is reversed for medications (prodrugs) that require enzymatic activation; poor metabolizers are unable to produce substantial quantities of bioactive drugs and rapid metabolizers produce too much. The optimum use of therapeutic drugs requires some knowledge of enzyme catabolic rates and how it affects the activation, inactivation, or clearance. The relationship between enzyme activity and pharmacogenomics has been extensively reviewed, e.g., the reader is referred to a recent Laboratory Medicine Practice Guidelines prepared by the National Academy of Clinical Biochemistry (NACB) [1].

## 2. Phenotype assessments and genotype classification schemes

In a living person, it is difficult if not impossible to determine the phenotype of an individual for a specific hepatic enzyme due to the inability to obtain liver tissue. With the exception of thiopurine methyltransferase, the analysis of activity from blood is not a surrogate of tissue enzyme activity, as it is only a reflection of tissue necrosis and turnover. Measurement of the rate of substrate metabolism and/or product formation is therefore used to the assess phenotypes. This requires the subject to have taken the drug, either in single dosage or at steady state. Accurate assessments also require that the subject be free of other influences such as co-medications that can induce or inhibit hepatic gene expression or enzyme activity. The analysis of the genetic makeup for metabolic genes is an alternate means to assess rates of metabolic capability. Analysis for germ-line polymorphisms has the advantage of not needing the individual to be on the drug itself. There have been schemes developed to correlate genotypes to phenotypes which are relevant for clinical pharmacogenomics.

The classical phenotype classification scheme for a specific drug is to compare individuals with variant activities against those who were considered normal or comprised of the majority of subjects, and are defined as the wildtype "extensive metabolizer." As shown in Table 1 among the variant genotypes are poor metabolizers (PM) associated with the presence of null genotypes, intermediate metabolizers (IM) with reduced metabolism genotypes, and ultra-rapid metabolizers (UM) as having gene duplications. This classical designation of metabolic states is rather simplified and does not explain all of the possible combinations of two variant alleles. Therefore the activity score was created which assigns for each allele, a value of 0 for null, 0.5 for intermediate, 1.0 for wildtype and 2 times these scores for the corresponding gene duplication genotypes [2]. This scoring scheme enables the classification of two additional intermediate states between PM and IM, and IM and EM (Table 1). Within this framework, the remainder of this review focuses on enzymatic rates for specific substrates that have current or near future relevance in clinical practices. Phenotype-genotype analyses for other enzyme-catalyzed metabolic pathways have been described, e.g., glutathione-S-transferase and the remethylation pathways, are herein not discussed.

**Table 1:** Association of enzyme metabolic rates to genotypes

Classical metabolism status	**Activity score**^**1**^	Genotypes
Poor metabolizer (PM)	0.0	Homozygous null gene
	0.5	Heterozygous null and reduced metabolism
Intermediate metabolizer (IM)	1.0	Homozygous reduced metabolism
		Heterozygous null and wildtype
	1.5	Heterozygous reduced metabolism and wildtype
Extensive metabolizer (EM)	2.0	Homozygous wildtype
		Heterozygous null and ultra metabolism
		Heterozygous reduced and ultra metabolism
Ultra-rapid metabolizer (UM)	> 2.0	Heterozygous wildtype and ultra metabolism
		Homozyogous ultra metabolism

^1^For each allele, a score of 0 is given for null genes, 0.5 for intermediate, and1.0 for extensive metabolizers. Alleles carrying gene duplications receive double the value compared to the assigned activity score with a single gene copy. The sum of both alleles is given.

## 3. Cytochrome P450 enzymes

The cytochrome enzymes currently consist of 57 enzymes containing different family and subfamily members and isoenzymes within each of them [1]. The most clinically relevant isoforms for pharmacogenomic practice are CYP 2D6, CYP 2C9, and CYP 2C19. Each of the major isoenzymes have genetic variants that may or may not affect catabolic rates. For many genes, there are a large number of variants. It is not practical or necessary to phenotypically characterize each variant, and only polymorphisms with the highest incidence and where data is available will be discussed.

### CYP 2D6

CYP 2D6 is also known as debrisoquine hydroxylase that catalyzes the oxidation of approximately a quarter of all the commonly therapeutic drugs in used clinical practice today. CYP 2D6 hydroxylates aromatic rings or an accompanying short side-chain of basic aryl-alkyl amines containing a protonated nitrogen. Of all enzymes of pharmacokinetic importance, the CYP 2D6 gene that encodes this enzyme has one of the highest numbers of genetic polymorphisms, with over 100 variants described to date [3]. However, many of these variants are rare and are not detected by routine CYP 2D6 single nucleotide polymorphism (SNP) assays. These variants can only be verified by sequencing of the CYP 2D6 gene. Studies indirectly characterizing the enzymatic activity of CYP 2D6 variants have used model drugs known to be effective substrates. In the model developed by Gaedigk et al., the ratio of dextromethorphan (DM) and its principal metabolite *o*-demethylated metabolite (DX) in a 4-hour urine collection was used following a single 0.3 mg/kg dose [4]. Using the DM/DX ratio, these investigators generalized that the PM was ≥0.3, IM between 0.03 and 0.3, EM between 0.03 and 0.0003, and UM < 0.0003. This indicates that relative to the wildtype, PM subjects had between 100 and 1000 times less enzyme activity, the IM subjects had between 10 and 100 times less activity, and UM had greater (sometimes 10 fold higher) enzyme activity. It should be noted that within a classification, there were significant overlap in the DM/DX ratio. While drugs are used to control specific and easily quantifiable symptoms, e.g., dextromethorphan is widely used as an antitussive, there may be no interest in performing routine clinical pharmacogenomic testing. Dosing and efficacy can be assess clinically, e.g., by the presence or absence of coughing.

In contrast, an analysis of CYP 2D6 polymorphism is important in the assessment of patients with breast cancer treated with the prodrug tamoxifen, a selective estrogen receptor modulator. Tamoxifen is activated through two pathways to the final product, endoxifen, which is the 10-100 fold more effective in blocking estrogen receptors than tamoxifen itself. The major pathway is first to N-desmethyltamoxifen (NDM) through CYP3A and then endoxifen via CYP 2D6. The minor pathway is to 4-hydroxytamoxifen through CYP 2D6, CYP 2B6, CYP 2C9, and CYP 2C19, and then to endoxifen through CYP 3A. In a study by Borges et al., the ratio of endoxifen to NDM was determined in a group of patients who were PM, IM, and EM [5]. The relative enzyme activity for the EM was roughly double that of the IM (0.15 vs. 0.08, respectively), which in turn was double that of the PM (0.08 vs. 0.04). The effect of genetic polymorphism for tamoxifen metabolism is considerably less than for dextromethorphan, due to the existence of alternate non-CYP 2D6-dependent metabolic pathways. Given that there is a minimum amount of endoxifen needed for therapeutic efficacy ("threshold" effect) [6], poor metabolizers produce an insufficient amount of drug to effectively block estrogen receptors, and other medications such as aromatase inhibitors are preferred [7]. The importance of CYP 2D6 genotypes for tamoxifen therapy for disease and relapse-free survival for breast cancer patients continues to be debated with studies showing both benefits [8] and no benefit [9].

The pharmacogenomics of the tricyclic antidepressants has been examined. The major metabolic pathway for amitriptyline is demethylation to the active metabolite nortriptyline by CYP 2C19. Thereafter, nortriptyline is deactivated by hydroxylation to 10-hydroxy metabolite by CYP 2D6. Steimer et al. compared the relative change in serum nortriptyline concentrations following 75 mg twice a day, as a function of variant CYP 2D6 alleles (*2, *4, *10, and *41) against the wildtype (*1) [10]. They found no significant difference in nortriptyline concentrations for *2 (thereafter the *1 and *2 groups were combined), a 40% (1.9-fold) decreased enzyme activity for *41, 63% (2.7-fold) decrease for *10, and a 96% (3.8-fold) decrease for *4. For combinations of variant alleles, the expected effect would be multiplicative, e.g., the *4/*41 genotype would have 173% reduced enzyme activities. Previously, the *10 and *41 alleles were associated with intermediate metabolism and the *4 allele was associated as a poor metabolizer [11]. These results are consistent with these classifications. The toxicities of tricyclic antidepressants are well described and develop gradually. Therefore, most psychiatric practices have not endorsed routine CYP 2D6 testing of their patients.

The enzyme reaction rate for the metabolism of codeine to morphine by *o*-demethylation by CYP 2D6 has been studied. Kirchheiner et al. examined the ratio of plasma area under the curve for morphine vs. codeine in relation to CYP 2D6 activity score [12]. They found that the extensive metabolizers (activity score 1.5 and 2.0) was 6 and 16-fold more active than the poor metabolizer (score 0.0), while the ultra metabolizer (3 full activity copies, score 3.0) had 50% more activity than the extensive metabolizers. Clinical pharmacogenomic testing is relevant for opiates, as morphine toxicity has been described in patients who are ultra-metabolizers of codeine [13]. Recently the FDA has issued a warning to physicians and consumers to be on the alert for unexpected signs and symptoms of morphine toxicity for patients with therapeutic dosing of codeine use. Nevertheless, the development of morphine toxicity is based on clinical presentation and routine screening for CYP 2D6 is impractical and expensive to the overall healthcare system, given the large number of subjects on opiate drugs.

### CYP 2C19

CYP 2C19 is also known as S-mephenytoin hydroxylase. CYP 2C19 acts on weakly or strongly basic drugs containing one hydrogen bond donor or if there are functional groups containing carbon or sulfur double bonded to oxygen present in the substrate. CYP 2C19 is responsible for the metabolism of anticonvulsant drugs, proton pump inhibitors, and drugs that inhibit platelet function. There have been no gene duplications described for the gene for this enzyme.

CYP 2C19 is a key enzyme in the activation of the clopidogrel, a widely used anti-platelet agent. Clopidogrel is metabolized first to 2-oxoclopidogrel and then to R-130964, the active metabolite that binds to the P2Y12 platelet receptor. There have been numerous reports demonstrating that individuals who are carriers for one or more of the poor function alleles for CYP 2C19, i.e., *2 or *3, have a higher incidence of adverse cardiac events such as acute myocardial infarction, cardiovascular death, or need for urgent revascularization [14,15]. The relative rate of metabolism for UM (CYP 2C19 *17), EM (*1/*1), IM (*1/*2 or *1/*3), and PM (*2/*2, *2/*3, or *3/*3) have been reported by Mega et al., who developed an assay for the active metabolite [15]. Using a 75 mg maintenance dose, individuals who had one copy of a null gene (IM) had a 30.7% lower area under the drug vs. time curve (AUC) vs. the wildtype, and those with two copies (PM) had a 45.6% lower AUC vs. the wildtype. The pharmacodynamic of a reduced metabolism genotype for clopidogrel is a decrease in the degree of platelet inhibition as measured by aggregation. This suggest therapeutic benefit with use of a higher dose, although there have been no published study to date that has documented the efficacy of increased dosing. Another option is to use an alternate platelet-inhibiting drug such as prasugrel, another prodrug but whose pharmacologic activity is not influenced by CYP 2C19 polymorphism [16].

The proton pump inhibitors (PPIs) are a class of compounds including omeprazole, lansoprazole, rabeprazole, esomeprazole, and pantoprazole, and are used to selectively and irreversibly inhibit gastric acid secretion. They have become essential in the treatment of patients with peptic ulcers, gastroesophageal reflux diseases and Zollinger-Ellison syndrome. They are also used for the eradication of *Helicobacter pylori *in combination with antibiotics. The PPIs metabolism by CYP 2C19. Relative to the wildtype, patients who are poor and intermediate metabolizers have an area under the omeprazole plasma concentration curve that is 6.8 and 3.0 times higher, respectively following 20 mg dosages [17]. Similar results are observed for rabeprazole at 5.3 and 1.7 times higher, respectively for these genotypes at 20 mg doses. The pharmacodynamic effect of the accumulation of PPIs due to reduced metabolism genotypes is a reduction in acid secretion, at least for omeprazole. Less drug is needed for therapeutic effectiveness among poor metabolizer patients. If through clinical trials pharmacogenomic testing can reduce the incidence of bleeding, testing routine testing for initial dosing will be justified.

### CYP 2C9

CYP 2C9 is another important member of the CYP P450 family responsible for metabolizing over 100 therapeutic drugs. The enzyme oxidizes neutral and acidic amphipatic drugs with a hydrophobic region. One hydrogen bond donor or anionic heteroatom is required. The relevant genotypes include *2 and *3, which are both associated with decreased enzyme activity. There are no CYP 2C9 gene duplications or deletions. The relative rate of enzyme activity has been described using 500 mg of the oral hypoglycemic drug tolbutamine as a model drug. The rate of metabolism to 4'-hydroxy-tolbutamide versus CYP 2C9 genotypes has been examined in healthy volunteers. Compared to the wildtype, individuals with intermediate metabolism (*1/*2 or *1/*3) had a 79% and 54% slower rate of metabolism, respectively [18]. Interestingly, the homozygous poor metabolizer (*2/*2) was only slightly lower at 52%.

While there is no clinical interest in tolbutamide for clinical pharmacogenomics, there is considerable interest in testing CYP 2C9 polymorphisms for S-warfarin dosing, especially when used in conjunction with demographic variables (age, gender, body weight) and genetic variances for vitamin K epoxide reductase complex subunit 1 (VKORC1). There are significance differences in the clearance rates for wildtype against CYP 2C9 variants. In one study, *1/*2 and *1/*3 heterozygous patients had an average of 45% lower clearance rate than the wildtype. The *2/*2 and *2/*3 variants had a 72% lower clearance rate, while the *3/*3 homozygous patient had an 88% lower clearance rate for warfarin than the wildtype [19]. The reduced clearance of the drug is related to the slower rate of enzyme activity for *2 and *3, estimated to be 12% and 5% of wildtype, for the *2 and *3 alleles, respectively [20]. Similar results have been reported for other oral anticoagulants. Thijssen et al. showed patients with the *1/*3 genotype had mean oral clearance rate that was 45% lower than the wildtype for acenocoumarol [21], and Ufer et al. reported 51% and 29% reduce clearance rates, respectively, for *2 and *3 for phenprocoumon [22]. For any of these drugs, there was no effect of CYP 2C19 polymormism on the pharmacokinetics of the less pharmacologically active R-isomers. Patients with reduce metabolism for CYP 2C9 require lower dosing than the standard 5 mg/day given to wildtype patients. The -1639 AA genotype for VKORC1 is also associated with a reduced warfarin dose requirement. The relative activities of other drug classes metabolized by CYP 2C19 such as nonsteroidal anti-inflammatory, antidiabetics, anticonvulsants, and angiotensin antagonists have also been reported [19].

## 4. Other metabolic enzymes

### Thiopurine methyltransferase (TPMT)

Thiopurine methyltransferase catalyzes the S-methylation of aromatic and heterocyclic sulfhydryl compounds such as mercaptopurine, and azathiopurine. These drugs are widely used to suppress the immune system for treatment of patients with autoimmune disease, leukemias and lymphomas, and following organ transplantation. Unlike the cytochrome P450 enzymes discussed above, genetic polymorphisms in TPMP are less frequent (8-10% for individuals heterozygous for *3A and *3C). When present, however, there are significant toxicological consequences to the affected individual, particularly related to hematopoietic dysfunction. TMPT is widely expressed including in erythrocytes in contrast to the cytochrome P450 enzymes which are located within the hepatocytes. Therefore the phenotype (enzyme activity) can be measured directly. Patients with the heterozygous intermediate genotype on the average had 1.7 times lower TPMP activities than the wildtype [23]. More significantly, those who have the poor metabolizer genotype for TMPT has 29 times lower enzyme activities than the wildtype and are particularly vulnerable to toxicity. Low activity alleles have been linked to a higher cure rate in childhood acute lymphocytic leukemia [24] but also a higher risk of second malignancies [25]. There is debate regarding the efficacy of genotyping vs. phenotyping for TPMT variances. Genotyping has an advantage in that it can be conducted in patients following blood tranfusions and is less technically demanding than the red blood cell enzymatic assay [26]. Phenotyping has the advantage of detecting rare variants that are not tested for by the targeted genetic assay. Irrespective as to which approach is taken, routine screening is warranted for patients on these medications given the significant and life-threatening consequences of inappropriate dosing.

### Uridine 5'-diphosphate (UDP)-glucuronyltransferase

UDP-glucuronyltransferase is a phase II enzyme that catalyzes the glucuronidation of various endogenous compounds such as bilirubin and steroids, and exogenous compounds such as drugs. The 1A1 isoenzyme acts on bilirubin and the colon cancer drug irinotecan, a prodrug that undergoes metabolism to the active metabolite SN-38 and then is inactivated via glucuronidation to SG-38N. The genetic variance in UDP 1A1 is characterized by the presence of additional TA repeats in the TATA sequence of the promoter gene. The wild type consists of 6 copies (*1). While 5 and 8 copies can occur, a common polymorphism is the presence of 7 TA repeats (*28). The enzymatic activity of individuals who are heterozyous (*1/*28) and homozygous (*28/*28) are 2.3 and 3.8 fold lower than the wildtype (*1/*1), respectively [27]. Accumulation of the SN-38 metabolite due to a decrease glucuronidation leads to gastrointestinal and hematopoietic toxicity. Lower doses of irinotecan and adding other chemotherapeutic agents such as *cis*-platin to the regimen would be indicated to treat these cancer patients to maintain efficacy and reduced side effects. Pharmacogenomic testing is indicated to avoid toxicities if drug doses are reduced in patients with variant genotypes.

## Summary

Understanding the relative enzyme activities for each genotype is only the first step in understanding the role of polymorphisms in the metabolism of relevant drugs. However, most drugs are metabolized my multiple enzyme pathways. Thus, even if an individual produces an inactive variant for an enzyme in the major pathway, the drug can be broken down through an alternate or minor pathway, assuming that these enzymes are not affected genetically. It is also likely that the mRNA expression of these genes from these individuals are up-regulated to compensate for the presence of the deficient enzyme. Therefore, estimations of reaction rates for a particular enzyme in isolation will overestimate the collective degree of metabolic activity. On the other hand, some individuals can simultaneously carry several null or reduced function alleles for multiple CYP genes. These individuals in particular will exhibit significant variances in the pharmacologic effect of the drugs relevant to their pharmacogenomic makeup.

The debate between the importance of genotyping versus phenotyping will continue for years to come with the weight of evidence for a particular drug favors one approach over the other. As these attributes are complementary, it is very likely that a combination of both will be useful in individualizing therapy for a given patient. For warfarin, genotying is important for initial dosing to avoid adverse events in initiation phase, but this does not supercede the need for therapeutic monitoring of anticoagulation. Should pharmacogenomic testing for tamoxifen become routine, identification of patients who are poor metabolizers may lead to an immediate decision to use an alternate medication. For wildtypes, tamoxifen can be administered to the premenopausal patient. Once steady state has been reached. therapeutic drug monitoring for endoxifen concentrations will be best in predicting outcomes given that the use of drug inhibitors can result in low metabolite levels. For clopidogrel, genotyping only affects the drugs pharmacokinetics. Testing for platelet function assess the drug's pharmacodynamic effect. A combination of genotyping and phenotyping may be important to make a medical decision of administering an alternate drug or increasing the dosage of clopidogrel itself. Clinical trials are needed to verify the value and economical justification of these tests. Thereafter, incorporation into clinical guidelines will facilitate physician education and acceptance.

## Competing interests

The author declares that they have no competing interests.
